# Association between sarcopenic obesity and mild cognitive impairment in patients with type 2 diabetes mellitus

**DOI:** 10.3389/fnut.2026.1778293

**Published:** 2026-06-16

**Authors:** Lei Zhang, Yonghong Cao, Yueyu Zhang, Wu Dai, Xin Wu, Jiajia Song, Dechao Yin, Xiaofang Han

**Affiliations:** 1 Department of Endocrinology, The Second People’s Hospital of Hefei, Hefei, Anhui, China; 2 Hefei Hospital Affiliated with Anhui Medical University, Hefei, Anhui, China; 3 Department of Neurology, The Second People’s Hospital of Hefei, Hefei, Anhui, China

**Keywords:** mild cognitive impairment, obesity, sarcopenia, sarcopenic obesity, type 2 diabetes mellitus

## Abstract

**Background:**

Type 2 diabetes mellitus (T2DM) is a major risk factor for cognitive decline. Sarcopenic obesity (SO), a metabolically detrimental trait, is common in people with T2DM. However, its association with mild cognitive impairment (MCI) remains poorly characterized.

**Methods:**

This study included 509 hospitalized patients aged ≥50 years with T2DM. Participants were classified into four groups: the normal group (N), the obesity-only group (O), the sarcopenia-only group (S), and the sarcopenic obesity group (SO). The Montreal Cognitive Assessment (MoCA) was used to evaluate MCI. Spearman’s correlation analysis and binary logistic regression were performed to examine the relationship between body composition and MCI. A sensitivity analysis was conducted to verify the robustness of the results.

**Results:**

The prevalence of MCI varied substantially among the groups, with the SO group having the highest at 76.0%, followed by the S group at 70.5%. The prevalence in both groups was markedly higher than that in the N group (45.3%) and the O group (53.3%). Correlation analysis revealed that the MoCA score was significantly positively correlated with the appendicular skeletal muscle mass index (ASMI) and inversely correlated with body fat percentage (BFP) (all *p* < 0.001). In a multivariable-adjusted binary logistic regression model, when the N group was used as a reference, being in the SO group (OR = 3.056) or S group (OR = 2.397) was identified as an independent risk factor for MCI, with the SO group demonstrating a more significant increase in risk. Further validated by modified Poisson regression, the risk of MCI was significantly elevated in the SO group (PR = 1.499) and the S group (PR = 1.401), whereas no statistically significant difference was observed in the O group. Subgroup analysis revealed that these relationships were more significant in patients aged 65 years and older and in female patients.

**Conclusion:**

Among middle-aged and older patients with T2DM, compared with sarcopenia or obesity alone, SO is a significant independent risk factor for MCI. Routine assessment of body composition is recommended in clinical practice to identify individuals at high risk of early cognitive decline.

## Introduction

Type 2 diabetes mellitus (T2DM) is a common metabolic disorder in middle-aged and older individuals. Persistent hyperglycemia not only leads to typical microvascular and macrovascular complications but also markedly increases the risk of cognitive decline and dementia (1). Prior research has demonstrated that individuals with T2DM have a 1.2–1.5-fold increased risk of mild cognitive impairment (MCI) (2) and a 1.5–2.0-fold heightened risk of Alzheimer’s disease (AD) (3), indicating the crucial involvement of metabolic irregularities in neurodegenerative mechanisms.

SO has attracted considerable attention in recent years as a consequence of T2DM. SO is characterized by a concurrent decrease in muscle mass and strength, accompanied by excessive fat accumulation, thus combining the adverse changes in the body composition of sarcopenia and obesity into a more harmful metabolic phenotype (4). The prevalence of SO is greater in the T2DM population and is strongly associated with exacerbated insulin resistance, increased inflammatory markers, mitochondrial dysfunction, impaired physical function, and reduced quality of life. Accumulating evidence indicates that SO may be a major contributor to cognitive deterioration in patients with T2DM. On the one hand, reduced myokine secretion caused by muscle atrophy and decreased physical activity leads to a decrease in brain-derived neurotrophic factor (BDNF), thereby impairing neuroplasticity (5, 6). On the other, obesity-associated chronic low-grade inflammation, adipokine dysregulation, and impaired insulin signaling pathways may accelerate gray matter atrophy, cerebral microvascular injury, and neurodegenerative changes (7).

The prevalence of SO varies greatly across populations because of the lack of standardized diagnostic criteria. Although clinical practice mainly follows the 2022 European Society for Clinical Nutrition and Metabolism/European Association for the Study of Obesity (ESPEN/EASO) consensus guidelines (8), these criteria are not fully applicable to the Asian population because of differences in body composition, genetic background, and muscle mass characteristics. Mo et al. reported that in elderly Chinese community-dwelling populations, the application of standardized diagnostic criteria for sarcopenia (15) combined with various obesity assessment methods – waist circumference (WC), body mass index (BMI), visceral fat area (VFA), and BFP – to define SO revealed discrepancies in its prevalence and diagnostic consistency (9). The diagnostic sensitivity of BMI combined with the AWGS criteria was the lowest, whereas the consistency of the other diagnostic approaches was relatively high. Recent studies have explored the association between SO and cognitive performance, but no consensus has been reached. A study involving 353 participants aged 40 and older revealed that those diagnosed with SO based on BMI and BFP exhibited the lowest overall cognitive function (10). Another study identifying SO through grip strength, BMI, and WC corroborated a significant relationship with diminished overall cognitive function (11). Related research in Japan indicated that individuals diagnosed with SO via grip strength and BMI faced a markedly elevated risk of MCI and dementia (12). Similarly, a Chinese study using the skeletal muscle index and BFP as diagnostic criteria also found an association between SO and an increased risk of cognitive impairment (13).

However, evidence regarding the strength, independence, and sex-related differences in the association between SO and cognitive impairment remains inconsistent. This is especially the case for middle-aged and elderly T2DM patients, a high-risk population for whom systematic epidemiological data are currently scarce. Therefore, this study aimed to systematically investigate the association between SO and MCI in middle-aged and older patients with T2DM to determine whether SO is an independent risk factor for cognitive decline and to explore potential sex differences and underlying metabolic mechanisms. These findings help elucidate the complex pathophysiological mechanisms underlying T2DM-related cognitive impairment and provide essential evidence for the early clinical identification of high-risk groups and the development of targeted body composition intervention strategies.

## Method

### Study population

**Figure 1:**
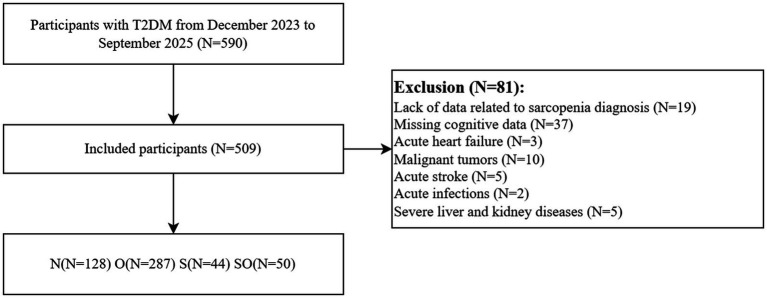
Flowchart.

This single-center, observational (non-interventional) cross-sectional study recruited hospitalized patients from the Department of Endocrinology, the Second People’s Hospital of Hefei, between December 2023 and September 2025, with study subjects selected via random sampling. The inclusion criteria were as follows: (a) met the 1999 WHO diagnostic criteria for diabetes mellitus (14); (b) were aged ≥ 50 years; (c) completed sarcopenia assessments (dual-energy X-ray absorptiometry (DXA), grip strength test, and 6-meter walk test) and an MCI assessment (MoCA); and (d) provided written informed consent. The exclusion criteria were as follows: (a) severe cardiopulmonary impairment requiring walking aids for independent movement, inability to complete the 6-meter walk test or grip strength assessment, or dependence on assistance for activities of daily living; (b) acute-phase stroke within 2 weeks of onset, or dementia caused by physical, chemical, or intracranial space-occupying lesions; (c) acute stress, concurrent trauma, infection, fever, oedema, or dehydration; (d) a history of hyperthyroidism or adrenal disorders (including Cushing’s syndrome and adrenal insufficiency), severe acute or chronic diabetic complications (including diabetic ketoacidosis and stage 5 chronic kidney disease), malignant tumors, or severe autoimmune diseases. All the participants understood the nature of the study and signed informed consent forms. This study was approved by the Clinical Trial Ethics Committee of the Second People’s Hospital of Hefei and was conducted in accordance with the Declaration of Helsinki (Figure 1).

### Clinical and biochemical analysis

Upon admission, venous blood samples were collected from all participants by endocrinology nurses on the morning after an overnight fast of at least 8 h. Key biochemical markers, including fasting blood glucose (FBG), glycated hemoglobin (HbA1c), alanine aminotransferase (ALT), and aspartate aminotransferase (AST), were measured. Serum concentrations of fasting insulin (FINS) and fasting C-peptide (FCP) were also measured. The homeostatic model assessment of insulin resistance (HOMA-IR) was calculated using the steady-state model assessment formula: HOMA-IR = fasting insulin (μIU/mL) × FBG (mmol/L)/22.5. Funduscopic examinations and electromyography (EMG) assessments were performed by experienced ophthalmologists and EMG technicians from our institution.

### Diagnostic criteria for T2DM

T2DM was diagnosed on the basis of the 1999 WHO diagnostic criteria for diabetes mellitus as follows (14): (1) presence of typical diabetic symptoms (polydipsia, polyphagia, polyuria, and unexplained weight loss) plus a random venous plasma glucose concentration > 11.1 mmol/L; (2) fasting venous plasma glucose concentration > 7.0 mmol/L (after a fast of at least 8 h); and (3) 2-h post-prandial plasma glucose concentration ≥ 11.1 mmol/L following oral administration of 75 g of anhydrous glucose. For participants without typical diabetic symptoms, a repeat test on a different day was required to confirm the diagnosis. T2DM was definitively diagnosed if at least one of the three criteria was met, and disease progression, HbA1c levels, islet β-cell autoantibodies, islet β-cell function, and other relevant assessments were evaluated.

### Diagnostic criteria for SO

Sarcopenia was diagnosed according to the 2019 AWGS criteria (15): An individual was diagnosed with sarcopenia if they had reduced skeletal muscle mass accompanied by decreased grip strength or impaired physical function. (1) Skeletal muscle mass: Appendicular skeletal muscle mass was measured using DXA (GE Lunar), and the ASMI was calculated as appendicular skeletal muscle mass (kg) divided by height squared (m^2^). An ASMI < 7.0 kg/m^2^ in male patients and <5.4 kg/m^2^ in female patients indicated skeletal muscle mass loss. (2) Grip strength: During measurement, participants were seated with their forearms resting on the armrests or at a 90° angle to their upper arms. Participants were instructed to squeeze a hand dynamometer (Xiangshan EH101) with maximal force. Each hand was tested twice, and the highest value was recorded. Decreased grip strength was defined as <28 kg in male participants and <18 kg in female participants. (3) Physical performance: A 6-meter straight distance was marked on a flat surface, with a 1-meter buffer zone at both ends. Participants were instructed to walk at their usual walking pace, and the time taken was recorded with a stopwatch. The average time of the two trials was calculated as the gait speed. Impaired physical function was defined as a gait speed < 1.0 m/s. Obesity was diagnosed on the basis of the WHO criteria as follows (16–18): BFP, measured via DXA, ≥25% in male participants and ≥35% in female participants. Participants were categorized into four groups on the basis of the diagnostic criteria for obesity and sarcopenia: normal (N, no obesity or sarcopenia), obesity-only (O, obesity without sarcopenia), sarcopenia-only (S, sarcopenia without obesity), and sarcopenic obesity (SO, both sarcopenia and obesity).

### Diagnostic criteria for MCI

The MoCA was used to assess global cognitive function. This tool has high sensitivity and specificity for identifying MCI and AD. The MoCA evaluates multiple cognitive domains, including attention, executive function, memory, language, visuospatial ability, abstract thinking, arithmetic, and orientation. MCI was defined according to the following criteria (19, 20): ≤13 points for illiterate participants, ≤19 points for participants with an elementary school education, and ≤24 points for participants with a junior high school education or higher.

### Statistical analysis

All statistical analyses were performed using SPSS 25.0. Kolmogorov–Smirnov tests were applied to all the quantitative data, baseline characteristics of continuous variables were expressed as mean ± SD deviation or interquartile ranges [*M*(P25, P75)], and categorical data are presented as percentages [*n* (%)]. One-way analysis of variance (ANOVA) was used for between-group comparisons of normally distributed data, while the Kruskal–Wallis *H* test was utilized for those with non-normal distribution. Categorical data were analyzed using the chi-square test. Multicollinearity was evaluated via the variance inflation factor (VIF). Spearman’s rank correlation analysis was used to examine the associations between MoCA scores and selected body composition parameters. Binary logistic regression analysis was used to assess the association between different body composition phenotypes and MCI. Stratified subgroup analyses by age and sex were conducted, and interaction effects were assessed. In this study, Little’s MCAR test was used to examine the missing data pattern, and the results (*p* > 0.05) indicated that the data were missing completely at random (MCAR). Only a small number of missing values were observed for HbA1c (0.98%), FCP (0.39%), and albumin (0.20%), with no missing values for the other variables. The expectation–maximization (EM) algorithm was used for the imputation of missing values, and a sensitivity analysis was conducted by re-running the multivariable logistic regression after excluding cases with missing values to verify the stability of the results (see Supplementary Table S2). A two-sided *p*-value of < 0.05 was considered to indicate statistical significance.

## Results

### Baseline characteristics

This study included 509 patients with T2DM, of whom 41.3% (*n* = 210) were male participants and 58.7% (*n* = 299) were female participants. Participants were classified into four groups according to the diagnostic criteria for obesity and sarcopenia: the N group (*n* = 128), O group (*n* = 287), S group (*n* = 44), and SO group (*n* = 50). Baseline characteristics (Table 1) revealed significant differences among the four groups. Participants in the SO group were significantly older (69.64 ± 7.86 years) and exhibited the worst physical performance, as reflected by the lowest grip strength (21.42 ± 6.38 kg) and slowest gait speed (0.85 ± 0.23 m/s) (all *p* < 0.001). Regarding body composition, the S group had the lowest ASMI (5.57 ± 0.82 kg/m^2^), whereas the obesity group presented the HOMA-IR [16.59 (7.78, 27.96)] and FINS levels (all *p* < 0.001).

**Table 1:** Baseline characteristics of the study population.

Variables	N (*n* = 128)	O (*n* = 287)	S (*n* = 44)	SO (*n* = 50)	*p*
Age (years)	62.72 ± 7.76	64.52 ± 8.40	67.75 ± 9.71	69.64 ± 7.86	<0.001
Female (%)	84 (65.66)	171 (59.58)	26 (59.09)	18 (36.00)	0.004
Diabetes duration (years)	10.00 (5.00, 16.00)	10.00 (5.00, 14.00)	12.00 (7.25, 18.00)	10.50 (4.00, 19.00)	0.117
BMI (kg/m^2^)	22.50 ± 2.06	26.05 ± 3.05	20.98 ± 1.93	23.47 ± 1.87	<0.001
BFP (%)	28.37 ± 5.61	36.19 ± 5.74	27.50 ± 6.16	34.50 ± 4.67	<0.001
WC (cm)	85.00 (79.13, 89.00)	92.50 (87.00, 99.00)	80.50 (75.25, 86.00)	89.50 (86.75, 94.50)	<0.001
ASMI (kg/m^2^)	6.36 ± 0.80	6.76 ± 0.99	5.57 ± 0.82	5.94 ± 0.66	<0.001
Grip strength (kg)	26.77 ± 9.64	26.80 ± 10.01	23.54 ± 12.22	21.42 ± 6.38	0.001
Gait speed (m/s)	1.12 ± 0.23	1.06 ± 0.28	0.86 ± 0.18	0.85 ± 0.23	<0.001
MoCA (points)	21.17 ± 5.48	21.19 ± 5.78	19.30 ± 6.48	19.16 ± 6.36	0.035
HbA1c (%)	8.68 ± 1.98	8.66 ± 1.85	8.63 ± 2.45	8.83 ± 2.42	0.954
FBG (mmol/L)	7.56 ± 3.21	8.03 ± 3.07	7.46 ± 2.58	7.38 ± 2.71	0.264
FCP (ng/mL)	1.32 (0.81, 1.89)	1.75 (1.18, 2.51)	1.31 (1.06, 1.85)	1.69 (1.10, 2.42)	<0.001
FINS (pmol/L)	28.35 (15.42, 49.50)	46.06 (25.50, 77.10)	26.46 (13.34, 48.36)	33.52 (17.44, 68.18)	<0.001
HOMA-IR	1.51 (0.81, 2.71)	2.77 (1.30, 4.65)	1.36 (0.75, 2.87)	1.84 (0.78, 3.99)	<0.001
Cr (μmol/L)	60.90 (50.78, 74.05)	61.90 (50.50, 74.10)	64.40 (52.85, 83.40)	65.60 (53.15, 93.95)	0.173
ALB (g/L)	41.15 (38.73, 43.58)	41.40 (39.10, 43.70)	40.05 (36.03, 41.85)	40.30 (37.28, 42.50)	0.007
ALT (U/L)	15.95 (12.13, 20.45)	17.30 (13.00, 26.30)	14.00 (10.15, 21.00)	17.30 (9.98, 24.38)	0.010
AST (U/L)	17.50 (15.00, 22.30)	18.40 (15.00, 22.40)	17.30 (13.45, 23.50)	17.80 (14.00, 23.00)	0.476
Hypertension (%)	64 (50.00)	188 (65.51)	25 (56.82)	35 (70.00)	0.012
Ischaemic Stroke (%)	89 (69.53)	204 (71.08)	38 (86.36)	45 (90.00)	0.005
NAFLD (%)	39 (30.47)	162 (56.45)	5 (11.36)	23 (46.00)	<0.001
DN (%)	22 (17.18)	53 (18.47)	13 (29.55)	10 (20.00)	0.323
DR (%)	40 (31.25)	76 (26.48)	12 (27.27)	8 (16.00)	0.232
DPN (%)	105 (82.03)	226 (78.75)	37 (84.09)	43 (86.00)	0.556
Exercise (%)	75 (58.59)	141 (49.13)	26 (59.09)	21 (42.00)	0.110
MCI (%)	58 (45.31)	153 (53.31)	31 (70.45)	38 (76.00)	<0.001

BFP, body fat percentage; BMI, body mass index; WC, waist circumference; HbA1c, glycosylated hemoglobin; ASMI, appendicular skeletal muscle mass index; FCP, fasting C peptide; FINS, fasting serum insulin; FBG, fasting blood glucose; HOMA-IR, homeostasis model assessment 2-insulin resistance; Cr, creatinine; ALB, albumin; ALT, alanine transaminase; AST, aspartate aminotransferase; DR, diabetic retinopathy; DN, diabetic nephropathy; DPN, diabetic peripheral neuropathy; MCI, mild cognitive impairment.

Notably, cognitive function differed significantly across groups (*p* = 0.035), with the lowest MoCA scores observed in the S group (19.30 ± 6.48) and SO group (19.16 ± 6.36). Consistently, the prevalence of MCI was highest in the SO group (76.00%), followed by the S group (70.45%), both of which were significantly higher than that in the N group (45.31%) (*p* < 0.001). No significant intergroup differences were identified in HbA1c, diabetes duration, or AST levels (all *p* > 0.05).

*Post hoc* multiple comparisons with Bonferroni correction were conducted among the four groups. The results revealed significant differences in sex, age, ASMI, grip strength, gait speed, BFP, and WC across groups, while no significant differences were found in physical activity level or diabetes duration. Further pairwise comparisons showed significant differences in sex composition between the SO group and both the N and O groups. With respect to age, statistically significant differences were observed between the S and N groups, between the SO and N groups, and between the SO and O groups. Moreover, significant intergroup differences were detected in BMI, FCP, FINS, HOMA-IR, ALB, and NAFLD. For MCI-related outcomes, statistically significant differences were identified between the SO and N groups and between SO group and O groups (see Supplementary Table S3).

### Prevalence of MCI by sex and age group

**Figure 2:**
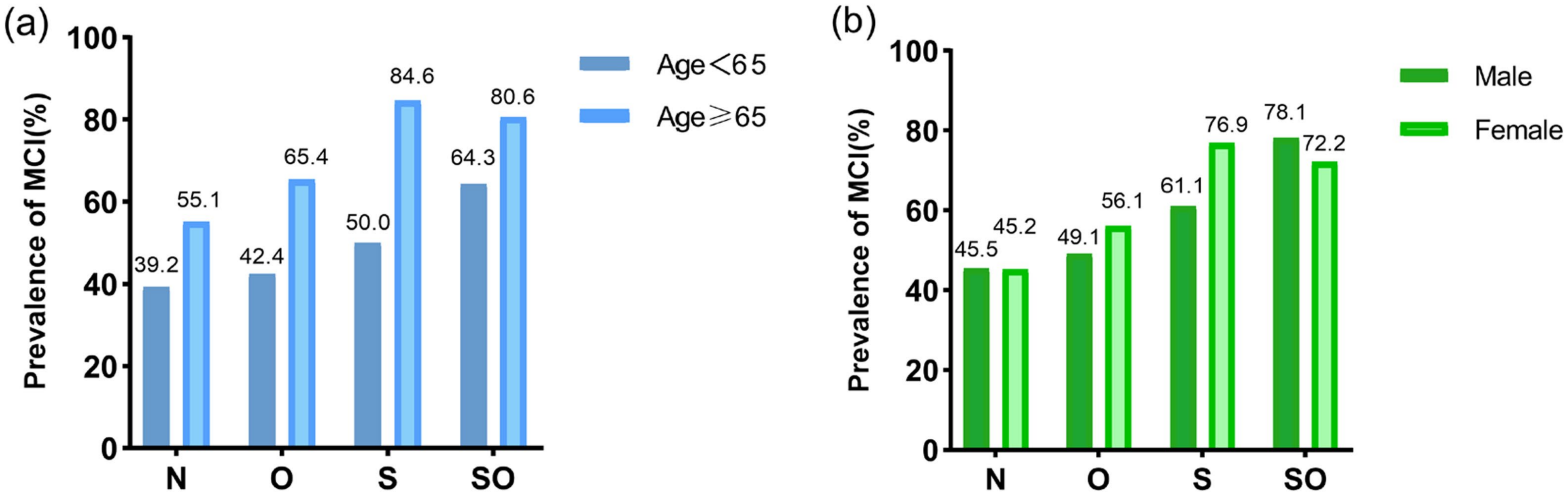
Prevalence of MCI by sex and age group. **(a)** Prevalence by age group (<65 vs ≥65 years); **(b)** Prevalence by sex (male vs female).

As shown in Figure 2a, the prevalence of MCI increased across subgroups in T2DM patients aged < 65 years, and was significantly higher in patients aged ≥ 65 years than in those aged < 65 years. The increasing trend in the prevalence of MCI across groups of male patients is shown in Figure 2b, with the highest prevalence (78.1%) observed in the SO group. In female patients, the prevalence of MCI in the sarcopenia-only group was 76.9%.

### Correlation between the MOCA score and various body composition variables

**Figure 3:**
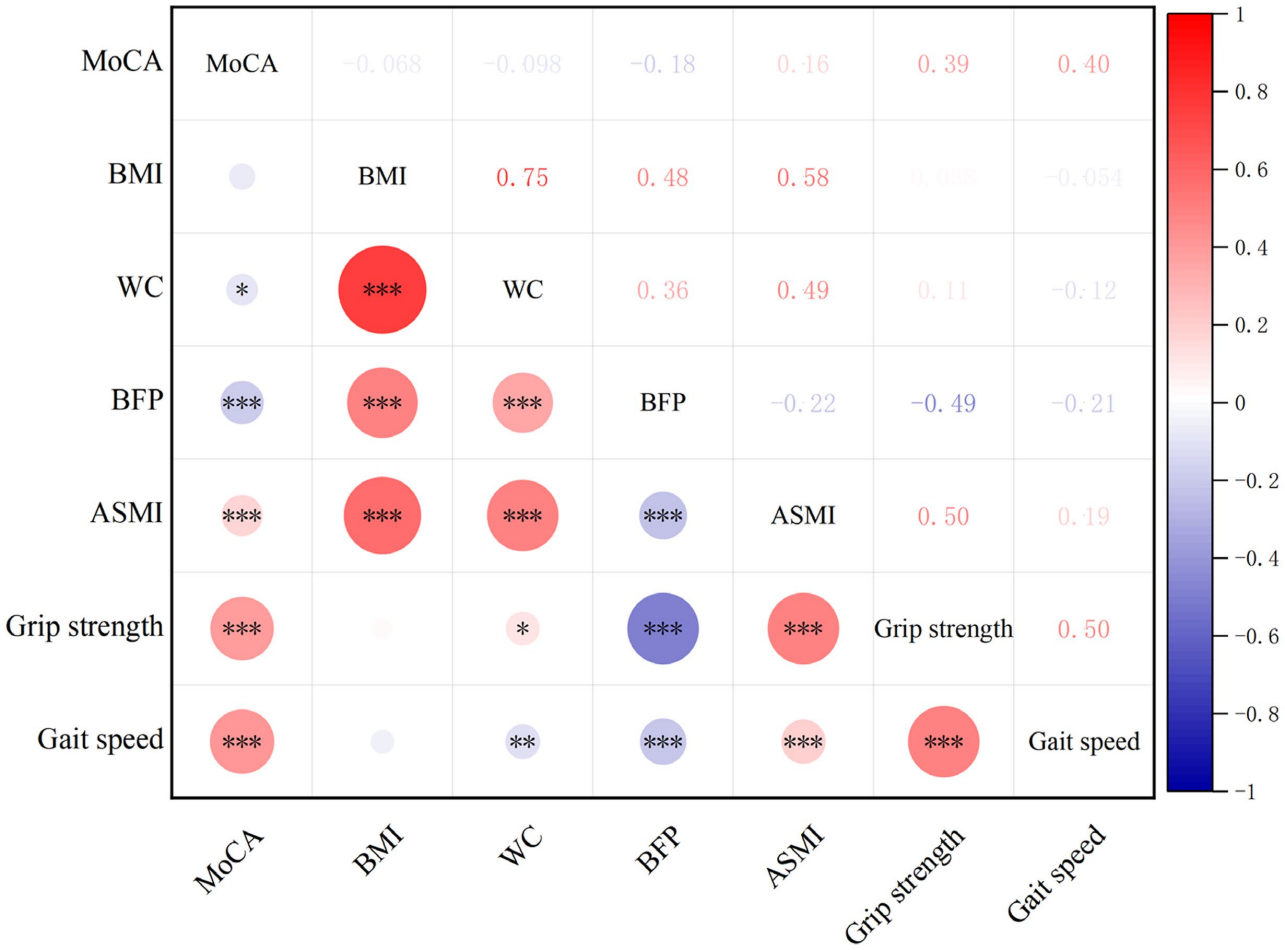
Correlations between the MOCA score and various body composition variables. BMI, body mass index; WC, waist circumference; BFP, body fat percentage; ASMI: appendicular skeletal muscle mass index. *p<=0.05, **p<=0.01, ***p<=0.001.

Spearman’s rank correlation analysis (Figure 3) revealed positive correlations among sarcopenia-related variables. The ASMI was positively correlated with grip strength (*r* = 0.50) and gait speed (both *p* < 0.001). BFP was significantly negatively correlated with the ASMI, grip strength, and gait speed (*p* < 0.001), indicating that a higher BFP is often associated with reduced muscle mass and strength. MoCA scores were negatively correlated with BFP (*p* < 0.001) and positively correlated with the ASMI, grip strength, and gait speed (all *p* < 0.001), indicating that both excessive body fat and muscle atrophy negatively affect cognitive performance.

### Binary logistic regression of associations between different body composition groups and cognitive impairment

Binary logistic regression analysis revealed that compared with the normal group (reference), the risks of MCI were significantly increased in the SO and sarcopenia-only (S) groups. The association was strongest in the SO group (OR = 3.822, 95% CI: 1.830–7.982, *p* < 0.001), followed by the S group (OR = 2.878, 95% CI: 1.380–6.003, *p* = 0.005). After adjustment for confounding factors including HbA1c, FCP, HOMA-IR, albumin, ALT, AST, and hypertension in model 3, the SO group remained an independent risk factor for MCI (OR = 3.056, 95% CI: 1.381–6.763, *p* = 0.006), with a higher risk than the S group (OR = 2.397, 95% CI: 1.106–5.196, *p* = 0.027). No significant association was observed between obesity-only and MCI. Sensitivity analysis after excluding missing values yielded consistent results: Obesity-only was not independently associated with MCI (*p* = 0.105), whereas the S group (OR = 2.776, 95% CI: 1.236–6.234, *p* = 0.013) and SO group (OR = 2.959, 95% CI: 1.327–6.595, *p* = 0.008) were identified as independent risk factors for MCI (see Supplementary Table S1 and Table 2).

**Table 2:** Binary logistic regression of associations between different body composition groups and cognitive impairment.

Variables	Model 1		Model 2		Model 3	
	OR (95% CI)	*P*	OR (95% CI)	*P*	OR (95% CI)	*P*
Normal	Ref.		Ref.		Ref.	
Obesity	1.378(0.907–2.094)	0.133	1.258(0.814–1.944)	0.301	1.419(0.896–2.247)	0.136
Sarcopenic	2.878(1.380–6.003)	0.005	2.335(1.082–5.008)	0.029	2.397(1.106–5.196)	0.027
Sarcopenic obesity	3.822(1.830–7.982)	<0.001	2.708(1.245–5.889)	0.012	3.056(1.381–6.763)	0.006

Model 1: Unadjusted.

Model 2: Adjusted for sex, age, diabetes duration, education level, and regular exercise.

Model 3: Adjusted for Model 2 plus HbA1c, FCP, insulin resistance, albumin, alanine, AST, hypertension, cerebral infarction, and fatty liver.

### Subgroup analysis and interactions

**Figure 4:**
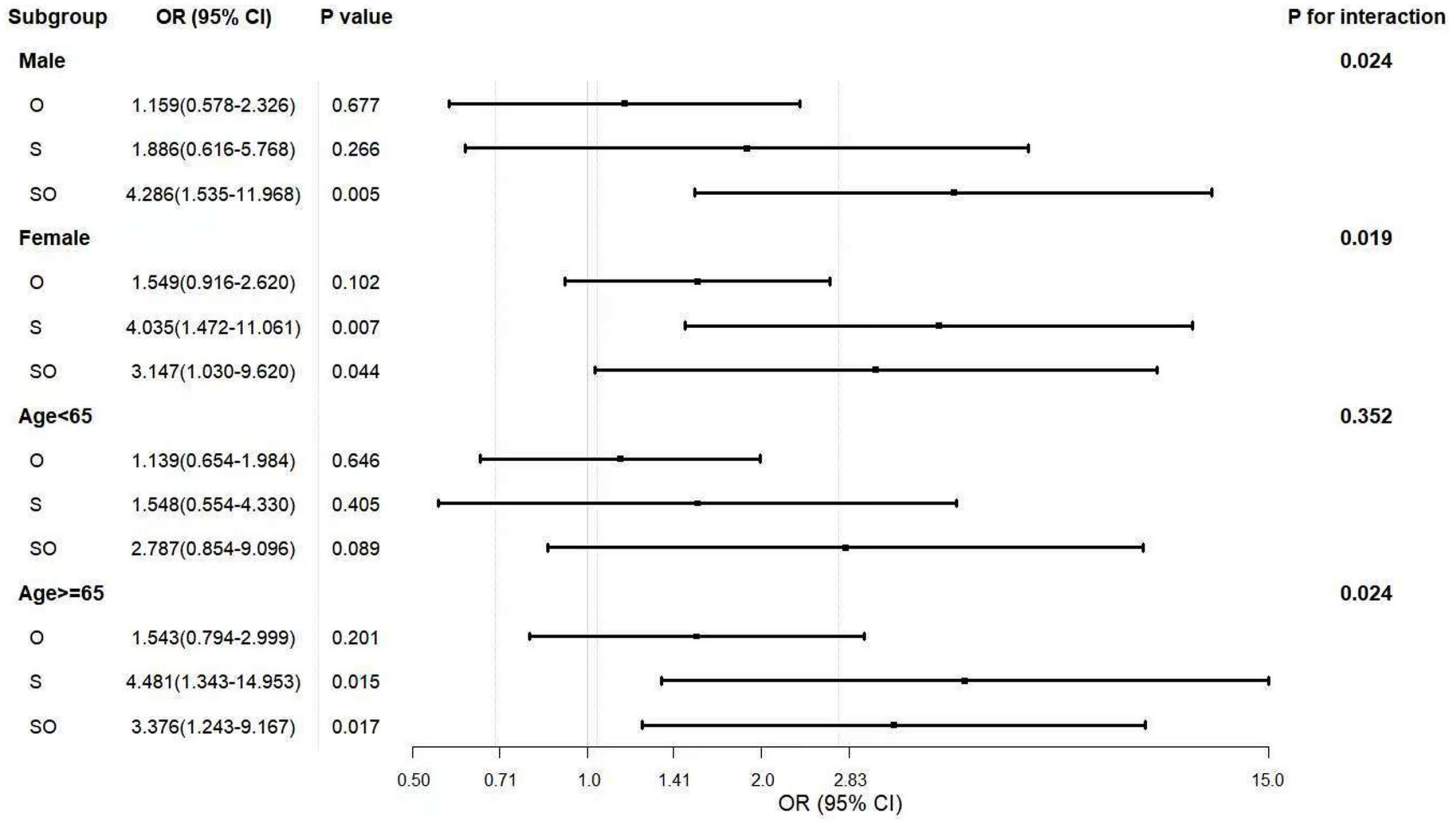
Subgroup analysis and interactions.

Subgroup exploratory analyses by sex and age indicated that only SO substantially increased the risk in men (*p* < 0.001), but both sarcopenia and SO considerably heightened the risk in female patients (*p* < 0.05). No substantial risk was detected for any trait in those under 65 years of age. Both sarcopenia and SO significantly elevated risk for those aged 65 years and older (*p* < 0.05). Notable interaction effects were detected for both sex and age (*p* < 0.05) (Figure 4).

### Sensitivity analysis

Sensitivity analysis was performed using modified Poisson regression to further verify the association between body composition phenotypes and MCI. Taking the normal group as the reference, the risk of MCI was significantly increased in the sarcopenia-only group (Model 3: PR = 1.401, 95% CI: 1.067–1.839, *p* = 0.015) and the SO group (Model 3: PR = 1.499, 95% CI: 1.147–1.959, *p* = 0.003) (Supplementary Table S5). No statistically significant difference was observed in the obesity-only group (*p* > 0.05). These findings were consistent with those of the aforementioned binary logistic regression, indicating the robustness and reliability of our conclusions.

## Discussion

The present study demonstrated that in middle-aged and older individuals with T2DM, compared with sarcopenia or obesity alone, SO is a more significant independent risk factor for MCI. These findings are consistent with those of prior studies. Someya et al. (12) analyzed data from 1,615 community-dwelling older persons in the Bunkyo Health Study and reported that, after controlling for confounding variables, obesity with sarcopenia was independently linked to MCI (OR = 2.11) and dementia (OR = 6.17). Booranasuksakul et al. (21) used NHANES cohort data from 1999 to 2002 and 2011 to 2014 to examine the relationships among body composition, SO, and cognitive performance in elderly individuals. The results demonstrated that the SO phenotype (OR = 1.9) and an elevated body fat/fat-free mass ratio (OR = 2.0) correlated with a heightened risk of cognitive impairment, with markedly worse cognitive test scores recorded in the high WC–low grip strength cohort (21). A study conducted by Wang et al. (13) with 945 community-dwelling Chinese older individuals reported a 6.0% prevalence of SO. Following comprehensive correction, SO (OR = 2.550) and obesity (OR = 2.141) separately increased the risk of cognitive impairment. SO, characterized by concurrent muscle loss and excess adiposity, may contribute to cognitive decline through multiple pathways. Adipose tissue in individuals with SO releases increased levels of proinflammatory mediators such as C-reactive protein (CRP) and tumor necrosis factor alpha (TNF-α), promoting systemic inflammation. These factors can cross the blood–brain barrier, directly damage neurons, promote apolipoprotein E synthesis, and exacerbate neurological impairment (22–24). Levine and Crimmins (25) reported that insulin resistance is a crucial mediator connecting SO to cognitive decline. This relationship may involve insulin-degrading enzymes (IDEs): elevated circulating insulin competes with IDEs, resulting in cerebral β-amyloid buildup and subsequent cognitive impairment and neurodegenerative alterations (26, 27). Sarcopenia indicates a reduction in motor performance, and because skeletal muscle is a significant endocrine organ, its atrophy diminishes the release of myokines such as irisin and BDNF, which possess anti-inflammatory and neuroprotective properties, thereby impairing cerebral protection (28). Patients in the SO group had elevated incidences of cerebral infarction, hypertension, and fatty liver disease, with vascular pathology serving as a principal cause of cognitive impairment in individuals with diabetes (29). Despite controlling for vascular risk variables, SO remained an independent factor, indicating that it may have further neurobiological impacts.

A Spearman’s correlation analysis indicated that the ASMI, grip strength, and walking speed were positively associated with the MoCA score; however, the BFP was negatively correlated with it. These results are consistent with those of earlier studies in geriatric populations and patients with diabetes (30–32). Multiple studies have shown that increased muscle mass, strength, and physical performance are frequently linked to enhanced cognitive ability (33, 34), but elevated body fat levels are associated with cognitive deterioration (35, 36). This study did not find a significant correlation between simple obesity and the likelihood of MCI. This outcome contrasts with those of certain studies, potentially because of the characteristics of the study population: the participants in this study were exclusively T2DM patients, who typically exhibited a pathological state of metabolic disorder and chronic inflammation, thereby obscuring the risk effect of simple obesity due to the underlying pathological alterations associated with diabetes. Subsequent analysis indicated that, upon controlling for obesity-related confounding variables such as insulin resistance and hypertension, the weak correlation between obesity and MCI further decreased. This implies that the influence of obesity on cognitive function is likely mediated through intermediary factors, such as metabolic irregularities and vascular damage, rather than through direct, distinct effects.

Subgroup analyses indicated substantial moderating effects of demographic variables on the relationship between body composition and MCI. Initially, concerning age, both sarcopenia and SO markedly increased the risk of MCI in T2DM patients aged ≥65 years, whereas no significant correlation was detected in those under 65 years. This may arise from older persons already facing age-related reductions in physiological reserves and cumulative pathogenic variables (37). The detrimental effects of sarcopenia and obesity increase susceptibility beyond the compensation threshold for cognitive function, resulting in notable clinical connections. Second, concerning sex, only SO emerged as a major risk factor for MCI in male patients, while both sarcopenia and SO were strongly correlated in women. This sex disparity may be associated with the reduction in estrogen levels in postmenopausal women. Estrogen provides preventive benefits for the neurological and musculoskeletal systems, and its sudden decline may increase women’s susceptibility to muscle loss (38, 39). Thus, even isolated sarcopenia may present considerable cognitive danger. Conversely, male androgens are intricately associated with muscle strength and synthesis, resulting in a very gradual and minor reduction. This finding highlights the further metabolic strain associated with obesity (40). These data indicate that preventative and intervention measures may require greater specificity. Senior male patients with T2DM should be especially cautious regarding the onset of SO, whereas senior female patients necessitate the concurrent consideration of both isolated sarcopenia and SO.

This study has several limitations. First, as a cross-sectional study, causality cannot be determined; prospective follow-up and interventional studies are needed to verify the findings. Second, this was a single-center study with relatively small sample sizes in the sarcopenia group and SO group, resulting in insufficient statistical power and wide confidence intervals in subgroup analyses. Therefore, the exploratory subgroup results regarding the differences in the associations of sarcopenia and SO with MCI across different sex and age strata should be interpreted with extreme caution, and overinterpretation or generalization to a broader population with T2DM should be avoided. Third, although the MoCA is a commonly used cognitive screening tool, it cannot replace a comprehensive neuropsychological evaluation. Moreover, the education-adjusted MoCA cut-off values used in this study, while validated in the general Chinese population, have not been specifically validated in patients with T2DM. Furthermore, diabetes-related metabolic abnormalities may affect cognitive performance and the accuracy of the screening tool, which could influence the results of the cognitive assessment to a certain extent, representing another important limitation of this study. In addition, data related to dietary habits, depressive status, medication use, and social support were not collected in this study. Thus, the potential confounding effects of these factors could not be adequately adjusted for in the statistical analyses, and residual confounding bias may therefore exist.

## Conclusion

This study revealed that in middle-aged and older people with T2DM, diminished muscle mass, compromised muscular strength, and lower walking speed are strongly correlated with cognitive impairment. Moreover, sarcopenia and SO exacerbate the likelihood of MCI, with age and sex serving as significant modifying factors. In contrast, obesity alone did not result in any supplementary cognitive risk. Therefore, SO should be recognized as a key phenotype for identifying patients at high risk of cognitive decline in the clinical management of T2DM, providing evidence for the development of targeted body composition interventions.

## Data Availability

The datasets presented in this article are not readily available because the dataset includes anonymized clinical data of diabetic patients, restricted by ethics for privacy protection and cannot be shared publicly per committee approval. Requests to access the datasets should be directed to Lei Zhang, zleijia2023@163.com.
