# Supplementary material for: Association between sarcopenic obesity and mild cognitive impairment in patients with type 2 diabetes mellitus
Source: Front Nutr. 2026 Jun 16;13:1778293. doi: 10.3389/fnut.2026.1778293 (PMC13314434; doi:10.3389/fnut.2026.1778293)
Supplement: Supplementary file 1 [file Data_Sheet_1.pdf]

Table S1: Sensitivity Analysis Logistic Regression Results After Deleting Missing Values

Variables	Model1		Model2		Model3	
	OR (95%CI)	P	OR (95%CI)	P	OR (95%CI)	P
Nomal	Ref.		Ref.		Ref.	
Obesity	1.41 (0.93 ~ 2.15)	0.107	1.305 (0.841 ~ 2.025)	0.235	1.471 (0.922 ~ 2.345)	0.105
Sarcopenic	3.13 (1.47 ~ 6.64)	0.003	2.506 (1.147 ~ 5.478)	0.021	2.776 (1.236 ~ 6.234)	0.013
Sarcopenic obesity	3.73 (1.78 ~ 7.82)	<.001	2.637 (1.204 ~ 5.776)	0.015	2.959 (1.327 ~ 6.595)	0.008

Model 1: Unadjusted

Model 2: Adjusted for gender, age, diabetes duration, education level, and regular exercise

Model 3: Adjusted for Model 2 plus HbA1c, fasting C-peptide, insulin resistance, albumin, alanine, aspartate aminotransferase, hypertension, cerebral infarction, and fatty liver

Table S2: Missing Value Analysis

Variable name	Total sample size	Missing value count	Missing value percentage (%)
Glycated hemoglobin (HbA1c)	509	5	0.98
Fasting C-peptide	509	2	0.39
Albumin (ALB)	509	1	0.20
Gender	509	0	0.00
Regular exercise	509	0	0.00
Education	509	0	0.00
Disease duration	509	0	0.00
Age	509	0	0.00
Insulin resistance	509	0	0.00
Alanine aminotransferase (ALT)	509	0	0.00
Peripheral vascular disease	509	0	0.00
Hypertension	509	0	0.00
Cerebral infarction	509	0	0.00
Fatty liver	509	0	0.00

Table S3 Multiple Comparison Analysis (Bonferroni)

Dependent Variable	(I) Group	(J) Group	Mean Difference (I-J)	Std. Error	Significance	95% Confidence Interval	
						Lower Bound	Upper Bound
sex	N	O	0.06043	0.05184	1	-0.0769	0.1977
		S	0.06534	0.08524	1	-0.1604	0.2911
		S+O	.29625*	0.08134	0.002	0.0808	0.5117
	O	N	-0.06043	0.05184	1	-0.1977	0.0769
		S	0.00491	0.07897	1	-0.2043	0.2141
		S+O	.23582*	0.07475	0.01	0.0378	0.4338
	S	N	-0.06534	0.08524	1	-0.2911	0.1604
		O	-0.00491	0.07897	1	-0.2141	0.2043
		S+O	0.23091	0.10082	0.135	-0.0361	0.498
	S+O	N	-.29625*	0.08134	0.002	-0.5117	-0.0808
		O	-.23582*	0.07475	0.01	-0.4338	-0.0378
		S	-0.23091	0.10082	0.135	-0.498	0.0361
age	N	O	-1.79693	0.88375	0.255	-4.1377	0.5438
		S	-5.03125*	1.45306	0.003	-8.88	-1.1825
		S+O	-6.92125*	1.38666	0	-10.5941	-3.2484
	O	N	1.79693	0.88375	0.255	-0.5438	4.1377
		S	-3.23432	1.34616	0.1	-6.7999	0.3312
		S+O	-5.12432*	1.2742	0	-8.4993	-1.7493
	S	N	5.03125*	1.45306	0.003	1.1825	8.88
		O	3.23432	1.34616	0.1	-0.3312	6.7999
		S+O	-1.89	1.71871	1	-6.4423	2.6623
	S+O	N	6.92125*	1.38666	0	3.2484	10.5941
		O	5.12432*	1.2742	0	1.7493	8.4993
		S	1.89	1.71871	1	-2.6623	6.4423
ASMI（kg/m2)	N	O	-.39379*	0.09587	0	-0.6477	-0.1399
		S	.79450*	0.15763	0	0.377	1.212
		S+O	.42277*	0.15043	0.031	0.0243	0.8212
	O	N	.39379*	0.09587	0	0.1399	0.6477
		S	1.18828*	0.14603	0	0.8015	1.5751
		S+O	.81655*	0.13823	0	0.4504	1.1827
	S	N	-.79450*	0.15763	0	-1.212	-0.377
		O	-1.18828*	0.14603	0	-1.5751	-0.8015
		S+O	-0.37173	0.18645	0.28	-0.8656	0.1221
	S+O	N	-.42277*	0.15043	0.031	-0.8212	-0.0243
		O	-.81655*	0.13823	0	-1.1827	-0.4504
		S	0.37173	0.18645	0.28	-0.1221	0.8656
Grip strength	N	O	-0.03289	1.0456	1	-2.8024	2.7366

		S	3.23168	1.71918	0.364	-1.3219	7.7853
		S+O	5.34831*	1.64062	0.007	1.0028	9.6938
	O	N	0.03289	1.0456	1	-2.7366	2.8024
		S	3.26457	1.5927	0.245	-0.954	7.4831
		S+O	5.38121*	1.50757	0.002	1.3881	9.3743
	S	N	-3.23168	1.71918	0.364	-7.7853	1.3219
		O	-3.26457	1.5927	0.245	-7.4831	0.954
		S+O	2.11664	2.03348	1	-3.2694	7.5027
	S+O	N	-5.34831*	1.64062	0.007	-9.6938	-1.0028
		O	-5.38121*	1.50757	0.002	-9.3743	-1.3881
		S	-2.11664	2.03348	1	-7.5027	3.2694
Gait speed	N	O	0.05461	0.02965	0.397	-0.024	0.1332
		S	.25679*	0.04857	0	0.1281	0.3855
		S+O	.27219*	0.04407	0	0.1554	0.389
	O	N	-0.05461	0.02965	0.397	-0.1332	0.024
		S	.20218*	0.04465	0	0.0839	0.3205
		S+O	.21758*	0.03971	0	0.1124	0.3228
	S	N	-.25679*	0.04857	0	-0.3855	-0.1281
		O	-.20218*	0.04465	0	-0.3205	-0.0839
		S+O	0.0154	0.05529	1	-0.1311	0.1619
	S+O	N	-.27219*	0.04407	0	-0.389	-0.1554
		O	-.21758*	0.03971	0	-0.3228	-0.1124
		S	-0.0154	0.05529	1	-0.1619	0.1311
PBF	N	O	-7.81767*	0.60034	0	-9.4078	-6.2275
		S	0.87415	0.98708	1	-1.7403	3.4886
		S+O	-6.13012*	0.94198	0	-8.6251	-3.6351
	O	N	7.81767*	0.60034	0	6.2275	9.4078
		S	8.69182*	0.91447	0	6.2697	11.114
		S+O	1.68755	0.86559	0.311	-0.6051	3.9802
	S	N	-0.87415	0.98708	1	-3.4886	1.7403
		O	-8.69182*	0.91447	0	-11.114	-6.2697
		S+O	-7.00427*	1.16755	0	-10.0967	-3.9118
	S+O	N	6.13012*	0.94198	0	3.6351	8.6251
		O	-1.68755	0.86559	0.311	-3.9802	0.6051
		S	7.00427*	1.16755	0	3.9118	10.0967
WC	N	O	-9.47755*	0.87329	0	-11.7906	-7.1645
		S	2.80625	1.43587	0.307	-0.9969	6.6094
		S+O	-6.19975*	1.37026	0	-9.8291	-2.5704
	O	N	9.47755*	0.87329	0	7.1645	11.7906
		S	12.28380*	1.33024	0	8.7604	15.8072
		S+O	3.2778	1.25913	0.057	-0.0573	6.6129

	S	N	-2.80625	1.43587	0.307	-6.6094	0.9969
		O	-12.28380*	1.33024	0	-15.8072	-8.7604
		S+O	-9.00600*	1.69838	0	-13.5045	-4.5075
	S+O	N	6.19975*	1.37026	0	2.5704	9.8291
		O	-3.2778	1.25913	0.057	-6.6129	0.0573
		S	9.00600*	1.69838	0	4.5075	13.5045
Exercise	N	O	0.09465	0.05301	0.449	-0.0457	0.235
		S	-0.00497	0.08715	1	-0.2358	0.2259
		S+O	0.16594	0.08317	0.279	-0.0544	0.3862
	O	N	-0.09465	0.05301	0.449	-0.235	0.0457
		S	-0.09962	0.08074	1	-0.3135	0.1142
		S+O	0.07129	0.07643	1	-0.1311	0.2737
	S	N	0.00497	0.08715	1	-0.2259	0.2358
		O	0.09962	0.08074	1	-0.1142	0.3135
		S+O	0.17091	0.10309	0.588	-0.1021	0.444
	S+O	N	-0.16594	0.08317	0.279	-0.3862	0.0544
		O	-0.07129	0.07643	1	-0.2737	0.1311
		S	-0.17091	0.10309	0.588	-0.444	0.1021
MoCA	N	O	-0.01976	0.61962	1	-1.6609	1.6214
		S	1.87642	1.01877	0.397	-0.822	4.5748
		S+O	2.01188	0.97222	0.234	-0.5632	4.587
	O	N	0.01976	0.61962	1	-1.6214	1.6609
		S	1.89618	0.94383	0.27	-0.6037	4.3961
		S+O	2.03164	0.89338	0.14	-0.3346	4.3979
	S	N	-1.87642	1.01877	0.397	-4.5748	0.822
		O	-1.89618	0.94383	0.27	-4.3961	0.6037
		S+O	0.13545	1.20503	1	-3.0563	3.3272
	S+O	N	-2.01187	0.97222	0.234	-4.587	0.5632
		O	-2.03164	0.89338	0.14	-4.3979	0.3346
		S	-0.13545	1.20503	1	-3.3272	3.0563
BMI	N	O	-3.53536*	0.33945	0	-4.4357	-2.635
		S	1.41141	0.57008	0.082	-0.1007	2.9235
		S+O	-1.09909	0.53347	0.24	-2.5141	0.3159
	O	N	3.53536*	0.33945	0	2.635	4.4357
		S	4.94677*	0.52438	0	3.5559	6.3376
		S+O	2.43627*	0.48433	0	1.1516	3.7209
	S	N	-1.41141	0.57008	0.082	-2.9235	0.1007
		O	-4.94677*	0.52438	0	-6.3376	-3.5559
		S+O	-2.51050*	0.66658	0.001	-4.2786	-0.7424
	S+O	N	1.09909	0.53347	0.24	-0.3159	2.5141
		O	-2.43627*	0.48433	0	-3.7209	-1.1516

		S	2.51050*	0.66658	0.001	0.7424	4.2786
Diabetes duration	N	O	0.80923	0.756	1	-1.1932	2.8117
		S	-2.03588	1.24302	0.612	-5.3283	1.2565
		S+O	-0.26856	1.18622	1	-3.4105	2.8734
	O	N	-0.80923	0.756	1	-2.8117	1.1932
		S	-2.84511	1.15157	0.083	-5.8953	0.2051
		S+O	-1.07779	1.09002	1	-3.9649	1.8093
	S	N	2.03588	1.24302	0.612	-1.2565	5.3283
		O	2.84511	1.15157	0.083	-0.2051	5.8953
		S+O	1.76732	1.47027	1	-2.127	5.6616
	S+O	N	0.26856	1.18622	1	-2.8734	3.4105
		O	1.07779	1.09002	1	-1.8093	3.9649
		S	-1.76732	1.47027	1	-5.6616	2.127
FCP	N	O	-.54737*	0.12442	0	-0.8769	-0.2178
		S	-0.02482	0.20457	1	-0.5667	0.517
		S+O	-.55891*	0.19523	0.026	-1.076	-0.0418
	O	N	.54737*	0.12442	0	0.2178	0.8769
		S	.52256*	0.18952	0.036	0.0206	1.0245
		S+O	-0.01153	0.17939	1	-0.4867	0.4636
	S	N	0.02482	0.20457	1	-0.517	0.5667
		O	-.52256*	0.18952	0.036	-1.0245	-0.0206
		S+O	-0.53409	0.24197	0.166	-1.175	0.1068
	S+O	N	.55891*	0.19523	0.026	0.0418	1.076
		O	0.01153	0.17939	1	-0.4636	0.4867
		S	0.53409	0.24197	0.166	-0.1068	1.175
FINS	N	O	-21.25573*	7.4033	0.026	-40.8648	-1.6467
		S	9.37904	12.17251	1	-22.8622	41.6203
		S+O	-11.69034	11.61629	1	-42.4583	19.0776
	O	N	21.25573*	7.4033	0.026	1.6467	40.8648
		S	30.63477*	11.27701	0.041	0.7654	60.5041
		S+O	9.56539	10.67422	1	-18.7073	37.8381
	S	N	-9.37904	12.17251	1	-41.6203	22.8622
		O	-30.63477*	11.27701	0.041	-60.5041	-0.7654
		S+O	-21.06938	14.39792	0.864	-59.2051	17.0663
	S+O	N	11.69034	11.61629	1	-19.0776	42.4583
		O	-9.56539	10.67422	1	-37.8381	18.7073
		S	21.06938	14.39792	0.864	-17.0663	59.2051
HbA1c	N	O	0.02584	0.21268	1	-0.5375	0.5892
		S	0.05256	0.34969	1	-0.8737	0.9788
		S+O	-0.14362	0.33371	1	-1.0275	0.7403
	O	N	-0.02584	0.21268	1	-0.5892	0.5375

		S	0.02672	0.32396	1	-0.8314	0.8848
		S+O	-0.16946	0.30664	1	-0.9817	0.6427
	S	N	-0.05256	0.34969	1	-0.9788	0.8737
		O	-0.02672	0.32396	1	-0.8848	0.8314
		S+O	-0.19618	0.41362	1	-1.2917	0.8994
	S+O	N	0.14362	0.33371	1	-0.7403	1.0275
		O	0.16946	0.30664	1	-0.6427	0.9817
		S	0.19618	0.41362	1	-0.8994	1.2917
FBG	N	O	-0.47277	0.32236	0.859	-1.3266	0.3811
		S	0.09731	0.53003	1	-1.3066	1.5012
		S+O	0.17797	0.50581	1	-1.1618	1.5177
	O	N	0.47277	0.32236	0.859	-0.3811	1.3266
		S	0.57007	0.49104	1	-0.7305	1.8707
		S+O	0.65074	0.46479	0.973	-0.5803	1.8818
	S	N	-0.09731	0.53003	1	-1.5012	1.3066
		O	-0.57007	0.49104	1	-1.8707	0.7305
		S+O	0.08066	0.62693	1	-1.5799	1.7412
	S+O	N	-0.17797	0.50581	1	-1.5177	1.1618
		O	-0.65074	0.46479	0.973	-1.8818	0.5803
		S	-0.08066	0.62693	1	-1.7412	1.5799
HOMA-IR	N	O	-8.20353*	2.85655	0.026	-15.7697	-0.6374
		S	3.75874	4.69675	1	-8.6815	16.199
		S+O	-3.26712	4.48213	1	-15.1389	8.6047
	O	N	8.20353*	2.85655	0.026	0.6374	15.7697
		S	11.96227*	4.35122	0.037	0.4372	23.4873
		S+O	4.93641	4.11863	1	-5.9726	15.8454
	S	N	-3.75874	4.69675	1	-16.199	8.6815
		O	-11.96227*	4.35122	0.037	-23.4873	-0.4372
		S+O	-7.02585	5.55542	1	-21.7404	7.6887
	S+O	N	3.26712	4.48213	1	-8.6047	15.1389
		O	-4.93641	4.11863	1	-15.8454	5.9726
		S	7.02585	5.55542	1	-7.6887	21.7404
Cr	N	O	0.33735	2.73528	1	-6.9076	7.5823
		S	-7.60213	4.49735	0.549	-19.5142	4.31
		S+O	-9.31231	4.29184	0.183	-20.6801	2.0555
	O	N	-0.33735	2.73528	1	-7.5823	6.9076
		S	-7.93948	4.16649	0.344	-18.9752	3.0963
		S+O	-9.64967	3.94378	0.089	-20.0955	0.7962
	S	N	7.60213	4.49735	0.549	-4.31	19.5142
		O	7.93948	4.16649	0.344	-3.0963	18.9752
		S+O	-1.71018	5.31957	1	-15.8001	12.3797

	S+O	N	9.31231	4.29184	0.183	-2.0555	20.6801
		O	9.64967	3.94378	0.089	-0.7962	20.0955
		S	1.71018	5.31957	1	-12.3797	15.8001
ALB	N	O	0.00912	0.43917	1	-1.1541	1.1723
		S	2.05838*	0.72208	0.027	0.1458	3.9709
		S+O	1.08756	0.68908	0.691	-0.7376	2.9127
	O	N	-0.00912	0.43917	1	-1.1723	1.1541
		S	2.04926*	0.66896	0.014	0.2774	3.8211
		S+O	1.07844	0.6332	0.535	-0.5987	2.7556
	S	N	-2.05838*	0.72208	0.027	-3.9709	-0.1458
		O	-2.04926*	0.66896	0.014	-3.8211	-0.2774
		S+O	-0.97082	0.85409	1	-3.233	1.2914
	S+O	N	-1.08756	0.68908	0.691	-2.9127	0.7376
		O	-1.07844	0.6332	0.535	-2.7556	0.5987
		S	0.97082	0.85409	1	-1.2914	3.233
ALT	N	O	-4.41077	1.80783	0.09	-9.1992	0.3776
		S	0.36868	2.97243	1	-7.5044	8.2417
		S+O	-0.55359	2.83661	1	-8.0669	6.9597
	O	N	4.41077	1.80783	0.09	-0.3776	9.1992
		S	4.77945	2.75376	0.499	-2.5144	12.0733
		S+O	3.85718	2.60656	0.837	-3.0468	10.7612
	S	N	-0.36868	2.97243	1	-8.2417	7.5044
		O	-4.77945	2.75376	0.499	-12.0733	2.5144
		S+O	-0.92227	3.51586	1	-10.2347	8.3902
	S+O	N	0.55359	2.83661	1	-6.9597	8.0669
		O	-3.85718	2.60656	0.837	-10.7612	3.0468
		S	0.92227	3.51586	1	-8.3902	10.2347
AST	N	O	-2.44676	1.35127	0.425	-6.0259	1.1323
		S	-0.99332	2.22176	1	-6.8781	4.8914
		S+O	-0.03369	2.12024	1	-5.6496	5.5822
	O	N	2.44676	1.35127	0.425	-1.1323	6.0259
		S	1.45344	2.05831	1	-3.9984	6.9053
		S+O	2.41307	1.94829	1	-2.7474	7.5735
	S	N	0.99332	2.22176	1	-4.8914	6.8781
		O	-1.45344	2.05831	1	-6.9053	3.9984
		S+O	0.95964	2.62795	1	-6.001	7.9203
	S+O	N	0.03369	2.12024	1	-5.5822	5.6496
		O	-2.41307	1.94829	1	-7.5735	2.7474
		S	-0.95964	2.62795	1	-7.9203	6.001
DPN	N	O	0.01837	0.0201	1	-0.0349	0.0716
		S	0.06747	0.03305	0.25	-0.0201	0.155

		S+O	-0.02344	0.03154	1	-0.107	0.0601
	O	N	-0.01837	0.0201	1	-0.0716	0.0349
		S	0.0491	0.03062	0.657	-0.032	0.1302
		S+O	-0.04181	0.02898	0.899	-0.1186	0.035
	S	N	-0.06747	0.03305	0.25	-0.155	0.0201
		O	-0.0491	0.03062	0.657	-0.1302	0.032
		S+O	-0.09091	0.03909	0.123	-0.1945	0.0126
	S+O	N	0.02344	0.03154	1	-0.0601	0.107
		O	0.04181	0.02898	0.899	-0.035	0.1186
		S	0.09091	0.03909	0.123	-0.0126	0.1945
NAFLD	N	O	-.25977*	0.05062	0	-0.3938	-0.1257
		S	0.19105	0.08322	0.133	-0.0294	0.4115
		S+O	-0.15531	0.07942	0.306	-0.3657	0.055
	O	N	.25977*	0.05062	0	0.1257	0.3938
		S	.45082*	0.0771	0	0.2466	0.655
		S+O	0.10446	0.07298	0.918	-0.0888	0.2978
	S	N	-0.19105	0.08322	0.133	-0.4115	0.0294
		O	-.45082*	0.0771	0	-0.655	-0.2466
		S+O	-.34636*	0.09844	0.003	-0.6071	-0.0856
	S+O	N	0.15531	0.07942	0.306	-0.055	0.3657
		O	-0.10446	0.07298	0.918	-0.2978	0.0888
		S	.34636*	0.09844	0.003	0.0856	0.6071
DN	N	O	-0.01279	0.04193	1	-0.1239	0.0983
		S	-0.12358	0.06894	0.442	-0.3062	0.059
		S+O	-0.02813	0.06579	1	-0.2024	0.1461
	O	N	0.01279	0.04193	1	-0.0983	0.1239
		S	-0.11079	0.06387	0.501	-0.28	0.0584
		S+O	-0.01533	0.06045	1	-0.1755	0.1448
	S	N	0.12358	0.06894	0.442	-0.059	0.3062
		O	0.11079	0.06387	0.501	-0.0584	0.28
		S+O	0.09545	0.08154	1	-0.1205	0.3114
	S+O	N	0.02813	0.06579	1	-0.1461	0.2024
		O	0.01533	0.06045	1	-0.1448	0.1755
		S	-0.09545	0.08154	1	-0.3114	0.1205
DR	N	O	0.04769	0.04702	1	-0.0768	0.1722
		S	0.03977	0.07731	1	-0.165	0.2445
		S+O	0.1525	0.07377	0.235	-0.0429	0.3479
	O	N	-0.04769	0.04702	1	-0.1722	0.0768
		S	-0.00792	0.07162	1	-0.1976	0.1818
		S+O	0.10481	0.06779	0.736	-0.0747	0.2844
	S	N	-0.03977	0.07731	1	-0.2445	0.165

		O	0.00792	0.07162	1	-0.1818	0.1976
		S+O	0.11273	0.09144	1	-0.1295	0.3549
	S+O	N	-0.1525	0.07377	0.235	-0.3479	0.0429
		O	-0.10481	0.06779	0.736	-0.2844	0.0747
		S	-0.11273	0.09144	1	-0.3549	0.1295
MCI	N	O	-0.07998	0.05212	0.753	-0.218	0.0581
		S	-.25142*	0.0857	0.021	-0.4784	-0.0244
		S+O	-.30687*	0.08178	0.001	-0.5235	-0.0903
	O	N	0.07998	0.05212	0.753	-0.0581	0.218
		S	-0.17144	0.07939	0.188	-0.3817	0.0388
		S+O	-.22690*	0.07515	0.016	-0.4259	-0.0279
	S	N	.25142*	0.0857	0.021	0.0244	0.4784
		O	0.17144	0.07939	0.188	-0.0388	0.3817
		S+O	-0.05545	0.10136	1	-0.3239	0.213
	S+O	N	.30688*	0.08178	0.001	0.0903	0.5235
		O	.22690*	0.07515	0.016	0.0279	0.4259
		S	0.05545	0.10136	1	-0.213	0.3239

The significance level for the mean difference is 0.05.

N: Normal population;S: Sarcopenia group;O: Obese group;S+O: Sarcopenia + obesity group;

BFP, body fat percentage; BMI, body mass index;WC, waist circumference;HbA1c, glycosylated hemoglobin; FCP, fasting C peptide;FINS, Fasting serum lisulin; FBG,fasting blood glucose;HOMA2-IR, homeostasis model assessment 2-insulin resistance;Cr, Creatinine;ALB, Albumin ;ALT, alanine transaminase; AST, aspartate aminotransferase; DR, diabetes retinopathy; DN, diabetes nephropathy; DPN, diabetic peripheral neuropathy;MCI mild cognitive impairment

Table S4. Multicollinearity Diagnostics for the Multivariate Model

Covariates (Model 3)	VIF	Tolerance
Sex	1.147	0.872
Age	1.499	0.667
Diabetes duration	1.197	0.835
Education	1.251	0.799
Regular exercise	1.056	0.947
Glycated hemoglobin (HbA1c)	1.130	0.885
Fasting C-peptide	1.280	0.781
Insulin resistance (HOMA-IR)	1.149	0.870
Albumin	1.087	0.920
Alanine transaminase (ALT)	5.655	0.177
Aspartate transaminase (AST)	5.409	0.185
Hypertension	1.092	0.915
Ischaemic stroke	1.270	0.787
Non-alcoholic fatty liver disease	1.099	0.910

Table S5. Modified Poisson regression analysis of the associations between different body composition groups and MCI

	Model 1		Model 2		Model 3	
Body Composition Groups	PR (95% CI)	P value	PR (95% CI)	P value	PR (95% CI)	P value
Normal	1.000 (Reference)	-	1.000 (Reference)	-	1.000 (Reference)	-
Obesity	1.176 (0.945-1.464)	0.146	1.123 (0.904-1.395)	0.293	1.177 (0.943-1.470)	0.149
Sarcopenia	1.555 (1.187-2.037)	0.001	1.380 (1.057-1.800)	0.018	1.401 (1.067-1.839)	0.015
Sarcopenic obesity	1.677 (1.312-2.145)	< 0.001	1.436 (1.104-1.868)	0.007	1.499 (1.147-1.959)	0.003

Note: PR, prevalence ratio; CI, confidence interval; MCI, mild cognitive impairment.

Model 1: Unadjusted crude model.

Model 2: Adjusted for sex, age, diabetes duration, education level, and regular exercise.

Model 3: Adjusted for variables in Model 2 plus glycated hemoglobin, fasting C-peptide, insulin resistance, albumin, alanine transaminase, aspartate transaminase, hypertension, ischaemic stroke, and non-alcoholic fatty liver disease.

Table S6. Normality test results of continuous variables

**Variable Name**	**Skewness**	**Kurtosis**	**Shapiro-Wilk (P value)**
Age (years)	0.402	2.494	< 0.001
ASMI (kg/m²)	0.684	4.099	< 0.001
Grip strength (kg)	1.151	5.934	< 0.001
Gait speed (m/s)	0.092	3.254	0.115
MoCA score	-0.455	2.392	< 0.001
Body mass index (kg/m²)	0.705	4.116	< 0.001
HbA1c (%)	0.963	3.996	< 0.001
Body fat percentage (%)	-0.324	2.688	< 0.001
Fasting blood glucose (mmol/L)	1.677	6.789	< 0.001
Diabetes duration (years)	0.539	2.937	< 0.001
Waist circumference (cm)	-0.437	7.209	< 0.001
Fasting C-peptide (ng/mL)	2.786	17.253	< 0.001
Fasting insulin (pmol/L)	5.581	45.349	< 0.001
HOMA-IR	5.625	45.616	< 0.001
Creatinine (μmol/L)	1.972	7.986	< 0.001
Albumin (g/L)	-1.052	20.940	< 0.001
ALT (U/L)	5.718	60.577	< 0.001
AST (U/L)	7.385	96.337	< 0.001
